# Incidence, prevalence and mortality of anorexia nervosa and bulimia nervosa

**DOI:** 10.1097/YCO.0000000000000739

**Published:** 2021-09-16

**Authors:** Annelies E. van Eeden, Daphne van Hoeken, Hans W. Hoek

**Affiliations:** a Parnassia Psychiatric Institute, The Hague, The Netherlands; b University of Groningen, University Medical Center Groningen, Department of Psychiatry, Groningen, The Netherlands; c Columbia University, Mailman School of Public Health, Department of Epidemiology, New York, New York, USA

**Keywords:** anorexia nervosa, bulimia nervosa, epidemiology, mortality

## Abstract

To review the recent literature on the epidemiology of anorexia nervosa and bulimia nervosa in terms of incidence, prevalence and mortality.

**Recent findings:**

Although the overall incidence rate of anorexia nervosa is considerably stable over the past decades, the incidence among younger persons (aged <15 years) has increased. It is unclear whether this reflects earlier detection or earlier age of onset. Nevertheless, it has implications for future research into risk factors and for prevention programs. For bulimia nervosa, there has been a decline in overall incidence rate over time. The lifetime prevalence rates of anorexia nervosa might be up to 4% among females and 0.3% among males. Regarding bulimia nervosa, up to 3% of females and more than 1% of males suffer from this disorder during their lifetime. While epidemiological studies in the past mainly focused on young females from Western countries, anorexia nervosa and bulimia nervosa are reported worldwide among males and females from all ages. Both eating disorders may carry a five or more times increased mortality risk.

**Summary:**

Anorexia nervosa and bulimia nervosa occur worldwide among females and males of all age groups and are associated with an increased mortality risk.

## INTRODUCTION

This review aims to provide an overview of the recently published studies on the epidemiology of anorexia nervosa and bulimia nervosa. It is an update of previous reviews on this subject in this journal [1–3]. For a review of the epidemiology of binge eating disorder, see Keski-Rahkonen in this issue [4]. 

**Box 1:**
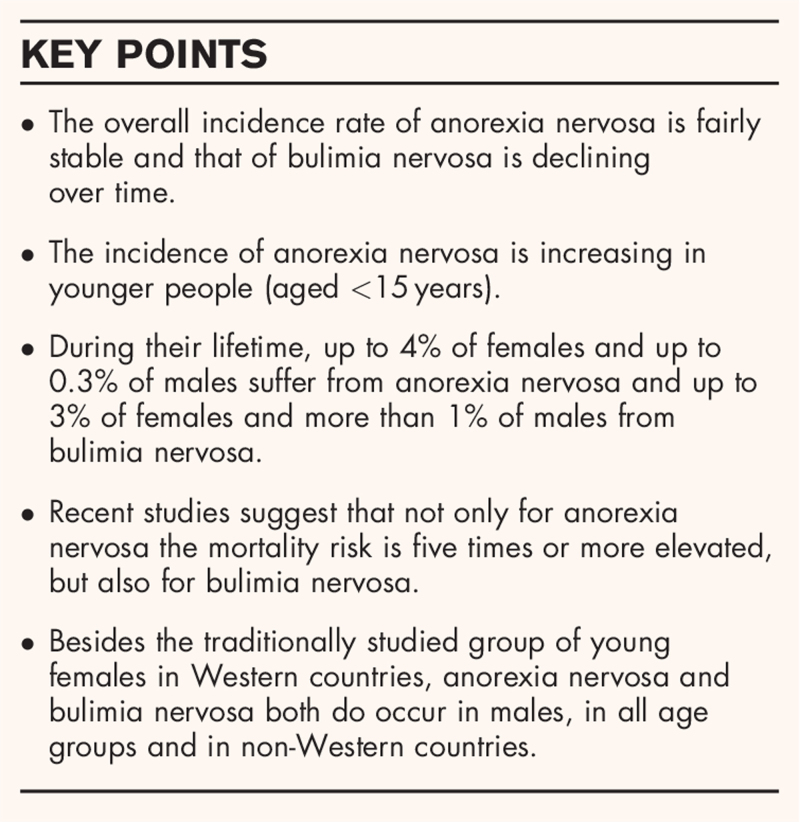
no caption available

Epidemiological studies provide information about the distribution (who, when and where) of disorders in a defined population and its trends over time. For eating disorders, there are some methodological problems regarding epidemiological research. Eating disorders are relatively rare in the community and help seeking is often avoided or delayed, for example for reasons of denial (particularly in anorexia nervosa) or stigma and shame (particularly in bulimia nervosa). These factors make general population studies on eating disorders costly and ineffective. Several strategies have been used to beat this problem, in particular the use of psychiatric case registers and other medical record-based studies. We must bear in mind that the results of these studies are an underestimation of the occurrence of eating disorders in the community, because not all patients will seek help, will be detected by their general practitioner or be referred to healthcare services. Moreover, different rates over time could be due to different case detection systems and diagnostic criteria, increased awareness leading to earlier detection and referral, and broader availability of treatment facilities, instead of a true increase in occurrence [1,5].

To review the literature, we searched for articles published in English using *Medline/PubMed*, *Embase, PsycINFO* and *Google Scholar,* using several key terms relating to epidemiology, anorexia nervosa and bulimia nervosa. We also checked the reference lists of the articles that we found for any additional articles missed by the database search.

## INCIDENCE

Incidence is the number of new cases of a disorder in a population over a specified period of time (usually 1 year). The incidence rate of eating disorders is commonly expressed per 100 000 persons per year (100 000 person-years). The study of newly developed cases of an eating disorder provides clues to unravel its etiology [6]. It is noteworthy that healthcare register-based incidence rates represent the situation at the moment of detection, which is likely to be later than the moment of disorder onset.

### Incidence of anorexia nervosa

Martinez-Gonzalez *et al.*[7^▪^] conducted a systematic review and meta-analysis of 31 published studies from 1980 to 2019 on the incidence of anorexia nervosa in females, mainly from Western countries. The incidence rates varied widely depending on the methodology, population and diagnostic criteria used. They reported on the incidence rates of only three population-based studies: 120 per 100 000 person-years among Swedish females aged 20–32 years [8], 200 per 100 000 person-years among Spanish females between the age of 12 and 22 years [9] and 270 per 100 000 person-years in Finnish twin females aged 15–19 years [10]. The pooled incidence rate of studies based on outpatient healthcare services (8.8 per 100 000 person-years; 95% confidence interval, CI: 7.8–9.8) was higher than that of hospital admissions (5.0; 95% CI 4.9–5.1) [7^▪^]. Compared to these all-age rates, the pooled incidence rates were higher for females aged 10–29 years, both in outpatient healthcare services [63.7 per 100 000 person-years (95% CI 61.2–66.1)] and in hospital admissions [8.1 (95% CI 7.6–8.5)] [7^▪^]. Furthermore, for healthcare register-based studies the incidence rates of anorexia nervosa showed a significant increase over time, especially in outpatient healthcare services. This does not necessarily mean a true increase in the occurrence of anorexia nervosa, as it could represent improved public awareness, detection and treatment rates over time.

Few studies have examined the incidence of anorexia nervosa in the general population; a limitation also encountered in the systematic review by Martinez-Gonzalez *et al.*[7^▪^]. The most recent study by Silen *et al.*[11^▪^] assessed the incidence rate of anorexia nervosa between the ages of 10 and 20 years according to the Diagnostic and Statistical Manual of Mental Disorders 5th Ed. (DSM-5) criteria in Finnish twins born between 1983 and 1987, yielding an incidence rate of anorexia nervosa of 320 (95% CI 230–440) per 100 000 person-years in the total group, 580 (95% CI 430–810) per 100 000 person-years among females and 30 (95% CI 10–310) per 100 000 person-years among males. These rates are higher than the rates in females described by Martinez-Gonzalez *et al.*[7^▪^]. This might be due to the use of the ‘broader’ DSM-5 criteria for anorexia nervosa by Silen *et al.*[11^▪^], and to the fact that twins may share genetic and environmental risk factors for anorexia nervosa, leading to a potential overestimation.

Population-based incidence rates are much higher than those derived from primary care and healthcare facilities, reflecting the selection filters on the pathways to (psychiatric) care [5]. Studies in general practices present incidence rates at the earliest stage of detection within the healthcare system. There have been no recent publications on all age groups in primary care since the previous review in this journal [1], that included a Dutch primary care study [12] which examined new cases with anorexia nervosa in a large representative sample of the Dutch population. The overall incidence rate of anorexia nervosa in Dutch females and males of all ages in primary care was fairly constant during three decades: in 1985–1989 it was 7.4 (95% CI 5.6–9.7) per 100 000 person-years, in 1995–1999 7.8 (95% CI 6.0–10.1) and in 2005–2009 6.0 (95% CI 4.3–8.1) [12]. Although no new studies have been published on incidence rates of anorexia nervosa in the total primary care population, Wood *et al.*[13^▪^] investigated 11- to 24-year-olds in England and found stable incidence rates between 2004 and 2014 (27.4 per 100 000 person-years; 95% CI 26.0–29.0). Demmler *et al.*[14] studied the incidence rate of eating disorders (all combined) in general practices in the UK since 2008, and reported a slight decline. The authors ascribe this finding to a decreasing trend in incidence of bulimia nervosa and a stable level of anorexia nervosa incidence, but they did not report rates per eating disorder diagnosis. Another primary care study among 10- to 19-year-olds in the UK reported an increased incidence rate in the age group of 13–16 years for all eating disorders combined, comparing 2018 rates to those in 2003 [15]. It is unclear whether this reflects a true increase in incidence or a shifting in age at detection.

The study of Reas and Ro [16^▪^], which was included in the meta-analysis by Martinez-Gonzalez *et al.*[7^▪^], described recent time trends in the incidence of anorexia nervosa in persons aged 10–49 years, using secondary care data of the Norwegian National Patient Register. The overall incidence rates for males and females combined were stable (differences nonsignificant) from 2010 to 2016, both for narrowly defined anorexia nervosa (18.8–20.4 per 100 000 person-years) and for broadly defined anorexia nervosa (33.2–39.5 per 100 000 person-years). They were also stable per sex for narrowly defined anorexia nervosa: in females 36.3–42.3 per 100 000 person-years, and in males 2.2–4.0 per 100 000 person-years; and for broadly defined anorexia nervosa: in females 63.3–79.1 per 100 000 person-years, and in males 4.4–5.9 per 100 000 person-years. The male to female ratio was found to be 1 : 13 for narrowly defined anorexia nervosa and 1 : 14 for broadly defined anorexia nervosa. This is in accordance with other studies showing considerably lower incidence rates for males, usually by more than a factor of 10, in comparison to females [11^▪^,^12^]. The incidence of anorexia nervosa according to DSM-5 criteria in 8- to 17-year-olds was examined in secondary care services in the UK and Ireland [17]. The overall incidence rate was 13.7 (95% CI 12.9–14.5) per 100 000 person-years; in females it was 25.7 (95% CI 24.1–27.3) and in males 2.3 (95% CI 1.8–2.8).

In females, the highest incidence rate of anorexia nervosa is around the age of 15 [11^▪^,^12^,^17^]. Several studies report a remarkable increase in the incidence of anorexia nervosa among girls aged 10–14 years [16^▪^,^17^]. Although most research has been performed in young females, some studies report incident anorexia nervosa cases in later life as well [12,16^▪^]. It is noteworthy that the peri-menopausal period has been suggested as another high-risk period in female life for the onset or recurrence of eating disorders [18,19]. In males, findings regarding the peak period of anorexia nervosa onset are less clear. While some studies have shown comparable [11^▪^] or a somewhat higher (age 16) peak age of onset [17], others found lower peak ages of 12–13 years [20] in comparison to females.

In summary, recent studies on time trends show fairly stable incidence rates for anorexia nervosa in the last decades [12,16^▪^], although some healthcare register-based studies suggest an increase in the incidence of anorexia nervosa [7^▪^], which might be explained by greater public awareness, better detection and the use of broader diagnostic criteria. Incidence rates in males are found to be lower, usually by more than a factor 10, in comparison to females [11^▪^,16^▪^]. The rates in males should be interpreted as an underestimation because of underdetection due to a double stigma: the stigma of having a psychiatric disorder, and an additional stigma of suffering from a ‘female-specific’ disorder [21]. Finally, the finding that the incidence of anorexia nervosa is increasing in younger girls (<15 years) has important implications for future research into risk factors [22], the development of prevention programs for younger subjects, and the planning of healthcare services.

### Incidence of bulimia nervosa

Few studies have investigated the incidence of bulimia nervosa. In the population cohort study of Finnish twins born in the 1980 s, the incidence rate of DSM-5 bulimia nervosa between 10 and 20 years of age was 100 (95% CI 60–190) per 100 000 person-years overall, and 180 (95% CI 110–340) per 100 000 person-years in females [11^▪^]. These population-based DSM-5 rates of bulimia nervosa are higher than DSM-IV rates for females aged 10–19 years identified in Dutch primary care (range 20.5–22.0 per 100 000 person-years) [12], partly because of the use of broader DSM-5 criteria in the Finnish study [11^▪^], but moreover because of the fact that only a small proportion of community ‘cases’ present to (primary) care [3,5]. It is of note that the Finnish study [11^▪^] investigated the incidence rate between 10 and 20 years of age, which only partly covers the peak age period of 15 to 29 years suggested by other studies [12,16^▪^].

The Dutch primary care study showed a significant decrease in the all-age incidence rate of bulimia nervosa according to DSM-IV criteria over three decades: in 1985–1989 it was 8.6 (95% CI 6.7–11.0), in 1995–1999 6.1 (95% CI 4.5–8.2) and in 2005–2009 3.2 (95% CI 2.0–4.9) per 100 000 person-years [12]. The English primary care study in 11- to 24-year-olds also showed a significant decline in the incidence rate of bulimia nervosa between 2004 and 2014 (incidence rate ratio: 0.5; 95% CI 0.3–0.7) [13^▪^]. Also, Demmler *et al.*[14] explained their all-age finding of a slight decrease in the incidence rate of all eating disorders combined in primary care to a declining trend in incidence of bulimia nervosa.

Findings of the Norwegian National Patient Register study [16^▪^] support a significant decline in overall incidence rates of bulimia nervosa in secondary care between 2010 and 2016, both in narrowly defined bulimia nervosa [2010: 18.5 per 100 000 person-years (95% CI 16.9–20.2); 2016: 16.1 (95% CI 14.6–17.2)] and in broadly defined bulimia nervosa (2010: 29.4 per 100 000 person-years (95% CI 27.4–31.5); 2016: 26.9 (95% CI 24.9–28.8). A significant decrease in incidence rates was found in all age groups, except for a trend of an increase among girls aged 10–14 years, suggesting a shift to earlier ages of onset or detection. The peak incidence was among females aged 20–29 years. In males, the incidence rates were low and stable over time, ranging between 0.9 and 1.6 for narrowly defined bulimia nervosa, and ranging between 1.7 and 2.5 for broadly defined bulimia nervosa. The male to female ratio was found to be 1 : 24 for narrowly defined bulimia nervosa and 1 : 26 for broadly defined bulimia nervosa. Incident cases of bulimia nervosa also occur in later life [12,16^▪^].

In conclusion, there is a decline in incidence rates of bulimia nervosa over time. The peak age of incidence ranged between 15 and 29 years. Studies of incidence in males are scarce, but the rates are found to be much lower than in females.

### Incidence of anorexia nervosa and bulimia nervosa in non-Western countries

Most epidemiological studies on eating disorders have been conducted in Western countries. Although studies assessing the epidemiology of eating disorders in non-Western countries are still scarce, we will highlight the most recent findings. Two Taiwanese studies [23^▪^,^24^] used national health insurance claim data to investigate the epidemiology of eating disorders between 2001 and 2013 in 10- to 49-year-old persons. In comparison to Western countries, the overall anorexia nervosa incidence rate in Taiwan is very low (1.1–1.3 per 100 000 person-years), but stable over time. Regarding bulimia nervosa, the incidence rates in Taiwan among females increased in the years up to 2009 [21.6 (95% CI 17.8–25.4) per 100 000 person-years] and then decreased [16.3 (95% CI 12.8–19.8) per 100 000 person-years] [24], following the trend in changes in incidence of Western countries, although a decade later. The incidence in Taiwan differs from that in Western countries in terms of older age at detection of anorexia nervosa and bulimia nervosa (20–29 years) and an increase in incidence among adults rather than adolescents [23^▪^,^24^]. This last finding aligns with findings of increasing numbers of persons in midlife with incident eating disorders in Western countries [19].

A study using data and methodology from the Global Burden of Disease (GBD) Study 2017 [25] reported increasing incidence rates of anorexia nervosa and bulimia nervosa from 1990 to 2017 in China, which is in contrast with the stable (anorexia nervosa) and decreasing (bulimia nervosa) rates in Western countries, but in line with the general trend of an increase in rates in all psychiatric diagnoses in China. Because of methodological issues related to the low prevalence and lack of global coverage of epidemiological studies for eating disorders, GBD calculations on these data must be interpreted with caution [26,27]. In this case [25], study results were difficult to check because 15 of the 39 publications from which data were included had been published in Chinese. From the abstracts of the included studies it would appear that in at least 26 of the 39 publications no formal eating disorder diagnosis had been applied, and that none of the studies addressed incidence. This casts doubts on the validity of the findings.

### Prevalence

Prevalence is the proportion of cases in a population present at a certain point or interval in time. The point prevalence is the prevalence at a specific date (point) in time. The 12-month prevalence is the prevalence over an interval of a year. The lifetime prevalence is the proportion of the population that has had the disorder at any moment in life up to the moment of registration. In general, lifetime prevalence rates are higher than point and 12-month prevalence rates, especially when assessed in older populations. Prevalence indicates the demand for care and is therefore useful in the planning of healthcare facilities. Many studies have assessed the prevalence of anorexia nervosa and bulimia nervosa. In this review, we focus on recently published systematic reviews and meta-analyses, supplemented by recently published population-based studies that have not been included in the described reviews and meta-analyses.

### Prevalence of anorexia nervosa

Galmiche *et al.*[28] have performed an extensive systematic review of 94 studies published between 2000 and 2018 that addressed the prevalence of formally diagnosed eating disorders in the general population. They explained the high variability of prevalence rates by the use of different diagnostic instruments [most commonly used: Structured Clinical Interview for DSM (13%), Composite International Diagnostic Interview (12%) and Eating Disorder Examination (11%)], diagnostic criteria [DSM-IV (78%), DSM-5 (14%) and DSM-III-R (4%)] and clinical investigation methods [face-to-face interview (51%), paper-and-pencil questionnaire (27%) or online or by telephone (22%)]. Weighted means were constructed from the prevalence rates and the population size of each study included, but were most likely not stratified for age. No confidence intervals were provided, only minimum-maximum ranges of the prevalence rates. The authors themselves conclude that the small number of studies makes it difficult to estimate weighted mean sex ratios. These limitations hamper the interpretation of some findings that are at odds with previous literature, in particular for point prevalence and 12-month prevalence rates. We therefore reproduce only the ranges of lifetime prevalence rates of anorexia nervosa, which were 0.1% to 3.6% in females and 0% to 0.3% in males.

Another systematic review and meta-analysis by Qian *et al.*[29] included 33 studies published between 1990 and 2020 on the prevalence of anorexia nervosa in the population. All studies combined, an overall lifetime prevalence rate of 0.2% (95% CI 0.06–0.3) was found. In studies applying DSM-5 criteria (18%; all in Western countries) a higher overall lifetime prevalence rate was found [0.9% (95% CI 0.7–1.1)]. This is in line with previous research showing an increase in anorexia nervosa prevalence rates when applying DSM-5 criteria, in comparison to rates according to DSM-IV criteria [30,31]. Lifetime prevalence rates in males and females (Table 1) were in the lower range of the rates described in the review of Galmiche *et al.*[28]. This could be explained by a large proportion of studies that had been conducted in Asia (40%) with very large sample sizes and much lower rates compared to Western countries, and the broader period studied (1984–2017) with especially lower prevalence rates before 2000 [29]. That is in contrast to findings described in a previous review in this journal [1] and to the notion that anorexia nervosa rates in Asian countries have been increasing in recent years and that they currently appear to be comparable to, or even higher than, those in Western countries [32–34]. In the review by Qian *et al.*[29], 6 of the 33 studies included had been published in Chinese and so the results were difficult to evaluate. However, there seems to be a lack of population-based studies using formal diagnostic interviews and applying DSM (-III-R, -IV, or -5) or International Classification of Diseases 10th Ed. (ICD-10) criteria. In Asia ‘non fat-phobic’ presentations of anorexia nervosa are common. In the DSM-5 the possibility of ‘persistent behaviour that interferes with weight gain’, which would apply to nonfat phobic presentations, is added to the DSM-IV B-criterium ‘intense fear of gaining weight or becoming fat’. Thus, the replacement of DSM-IV by DSM-5 criteria for anorexia nervosa will ultimately lead to higher rates among Asian people.

**Table 1:** Overview of recently published studies on prevalence rates. Studies are grouped by design and listed in chronological order

Study	Country	Study time	*N*	Age	Type of study	Criteria	Prevalence	Anorexia Nervosa	Bulimia Nervosa
*Systematic review*									
Lindvall Dahlgren *et al*., 2017 [35]	Worldwide	2012-2017	19 studies Range 496–22,397	11 and older	Two-stage design studies	DSM-5	Lifetime	♀ 1.7–3.6% ♂ 0.1% (1 study)	NR
							Point	♀ 0.7–1.2% ♂ 0.1% (1 study)	♀ 0.6% (2 studies)
					Interview-based studies	DSM-5	Lifetime	♀ 0.8–1.9%	♀ 2.6% (1 study)
					Self-report studies	DSM-5	Point	♀ 0.06–1.2%	♀ 0.5–8.7%
Galmiche *et al.,* 2019 [28]	Worldwide	2000-2018	94 studies Range 111–248,558	8 and older	Systematic review	DSM-III-R DSM-IV DSM-5	Lifetime	♀ 0.1–3.6% ♂ 0–0.3%	♀ 0.3–4.6% ♂ 0.1–1.3%
Qian *et al*., 2021 [29]	Worldwide	1984-2017	33 studies 315,877	15 and older	Systematic review and meta-analysis	DSM-III DSM-III-R DSM-IV DSM-5 ICD-10	Lifetime	♀ 0.6% (95% CI 0.3–1.1) ♂ 0.04% (95%CI 0.01–0.1)	♀ 1.2% (95% CI 0.7–1.9) ♂ 0.4% (95%CI 0.2–0.7)
						DSM-5	Lifetime	0.9% (95%CI 0.7–1.1)	1.4% (95%CI 0.0–6.3)
						DSM-III DSM-III-R DSM-IV DSM-5 ICD-10	12-month	♀ 0.03% (95%CI 0.0–0.06) ♂ 0.01% (95%CI 0.0–0.02)	♀ 0.3% (95%CI 0.1–0.6) ♂ 0.09% (95%CI 0.02–0.2)
						DSM-5	12-month	0.04% (95%CI 0.02–0.06)	0.1% (1 study)
*Two-stage design*									
Micali *et al*., 2017^a^[36^▪▪^]	UK	2009-2012	5,658	Mean: 47.8 (SD 4.5)	Two-stage design	DSM-5	Lifetime	♀ 3.6% (95%CI 2.8–4.7)	♀ 2.2% (95%CI 1.7–2.7)
							12-month	♀ 0.2% (95%CI 0.2–0.5)	♀ 0.4% (95%CI 0.2–0.7)
*Interview-based*									
Hay *et al*., 2017^b^[37]	Australia	2014-2015	5,737	15 and older	Interview-based	DSM-5	3-month	0.4–0.5%	1.1–1.2%
Udo and Grilo, 2018^a^^,^^b^[38^▪^]	USA	2012-2013	36,306	18 and older	Interview-based	DSM-5	Lifetime	♀ 1.4% (SE 0.1) ♂ 0.1% (SE 0.04)	♀ 0.5% (SE 0.06) ♂ 0.08% (SE 0.03)
							12-month	♀ 0.08% (SE 0.03) ♂ 0.01% (SE 0.01)	♀ 0.2% (SE 0.05) ♂ 0.05% (SE 0.02)
Chen *et al*., 2019 [39]	Taiwan	2015-2017	4,816	7-14	Interview-based	DSM-5	Lifetime	0.2% (95%CI 0.0–0.4)	NR
							6-month	0.2% (95%CI 0.0–0.4)	NR
Mohammadi *et al*., 2020 [40]	Iran	2016-2018	27,111	6-18	Interview-based	DSM-5	Lifetime	♀ 0.1% (95%CI 0.06–0.2)	♀ 0.1% (95%CI 0.07–0.2) ♂ 0.02% (95%CI 0.01–0.08)
Silen *et al.* 2020 [11^▪^]	Finland	2006-2009	1,347	Mean 22.4 (SD 0.7)	Interview-based	DSM-5	Lifetime	♀ 6.2% (95%CI 4.6–8.3) ♂ 0.3% (95%CI 0.08–1.3)	♀ 2.4% (95%CI 1.5–3.9) ♂ 0.2% (95%CI 0.02–1.1)
Bagaric *et al*., 2020^b^[47^▪▪^]	Australia	2017	2,977	Mean 53.9 (SD 19.1)	Interview-based	DSM-5	Lifetime	NR	♀ 2.6% (95%CI 2.1–3.2) ♂ 1.2% (95%CI 0.9–1.7)
							Point	NR	♀ 0.8% (95%CI 0.5–1.2) ♂ 0.4% (95%CI 0.2–0.7)
*Self-report*									
Ernst *et al*., 2017^a^[41]	Germany	2009	1,654	Mean: 13.4 (SD 5.8)	Self-report	DSM-5	Point	0.3% (95%CI 0.1–0.7)	0.4% (95%CI 0.2–0.8)
Glazer *et al*., 2019 [42]	USA	1996-2013	9,031	Mean at baseline: 11.6 (SD 1.6)	Self-report	DSM-5	Lifetime	♀ 1.6%	♀ 2.1%
Mitchison *et al*., 2020 [43]	Australia	2017	5,191	11-19	Self-report	DSM-5	Point	♀ 1.3% ♂ 0.0%	♀ 7.7% ♂ 1.8%

a included in Galmiche *et al*., 2019.

b included in Qian *et al*., 2021. CI, confidence interval; NR, not reported; SD, standard deviation; SE, standard error.

In their systematic review, Lindvall Dahlgren, Wisting and Ro [35] focused specifically on the prevalence of DSM-5 defined anorexia nervosa in the general population. Nineteen studies published until 2017 were included. Lifetime prevalence rates for anorexia nervosa in females differed according to the study method; 1.7–3.6% in studies with two-stage design and 0.8–1.9% in interview-based studies. Point prevalence rates in females ranged from 0.06% to 1.2%, predominantly assessed with self-reports.

Since this systematic review [35], several studies [11^▪^,^36^^▪▪^,^37^,38^▪^,^39^–^43^] have been published that investigated prevalence according to DSM-5 criteria. The prevalence rates reported in these recent studies are shown in Table 1 and are largely in line with those reported in the review. However, the population-based study of Finnish twins born in the 1980 s [11^▪^], in which the whole sample was diagnostically interviewed, found higher lifetime prevalence rates for anorexia nervosa: 6.2% (95% CI 4.6–8.3) in females and 0.3% (95% CI 0.08–1.3) in males. Suggested explanations for the higher rate found among females were the twin nature of the study, the thorough anorexia nervosa assessment and Finnish socio-cultural characteristics favouring a drive for thinness. Only 55% of the females identified with anorexia nervosa in the study reported that they had been diagnosed in real life by a healthcare professional.

Although epidemiological studies have mainly focused on the traditionally known high-risk group of young females, it has been shown in recent years that anorexia nervosa is prevalent among older persons as well [18,19]. The highest lifetime prevalence rates are found in adults, because of the accumulation of anorexia nervosa first emerging in the peak age period of adolescence [44] combined with incidence later in adulthood. A longitudinal, population-based study among Polish males showed that anorexia nervosa was prevalent in all age groups (10–80^+^ years) [45]. Although a decrease in point prevalence among females over a 30-year follow-up period was found, with no anorexia nervosa cases by age 50 [46^▪^], the 12-month prevalence rate in 40- to 50-year-old females was still 0.2% in another study [36^▪▪^].

### Prevalence of bulimia nervosa

The three previously discussed systematic reviews and meta-analyses [28,29,35] also reported prevalence rates for bulimia nervosa. The lifetime prevalence rates for bulimia nervosa ranged from 0.3% to 4.6% in females and from 0.1% to 1.3% in males in the extensive systematic review by Galmiche *et al.*[28]. As discussed previously, we refrain from reproducing the 12-month and point prevalence rates they report for methodological reasons.

Qian *et al.*[29] reported an overall lifetime prevalence rate for bulimia nervosa of 0.6% (95% CI 0.3–1.0). This review included a relatively large (40%) proportion of Asian studies. For bulimia nervosa the lifetime prevalence in Western countries was 7.3 times higher than that in Asian countries. The lifetime prevalence rates in females and males (Table 1) were close to the rates described by Galmiche *et al.*[28]. The pooled overall lifetime prevalence rate rose up to 1.4% (95% CI 0.0–6.3), when using only studies that applied DSM-5 criteria (18%; all in Western countries) [29]. Other studies supported this increase [30,31], which could be explained by the lower required frequency of binge eating and compensatory behaviour in DSM-5 compared to DSM-IV.

Lindvall Dahlgren *et al.* included only studies that had applied DSM-5 criteria and found few studies on bulimia nervosa, but the authors reported preliminary evidence for an increase in bulimia nervosa prevalence [35]. Two two-stage studies reported a point prevalence rate of 0.6% in females [30,48]. The lifetime prevalence rate was found to be 2.6% among females in one interview-based study [49]. In self-report studies, point prevalence rates ranged from 0.5% to 8.7% in females.

Since these systematic reviews, several studies on the prevalence of DSM-5 defined bulimia nervosa [11^▪^,^36^^▪▪^,^37^,38^▪^,^40^–^43^,^47^^▪▪^] have been published in recent years. Among females, lifetime prevalence rates around 2.3% (range 2.1–2.6%) were consistently found [11^▪^,^36^^▪▪^,^42^,^47^^▪▪^], which gives further support for an increase in bulimia nervosa prevalence since the introduction of DSM-5 criteria. Only one study in a large population-based sample of US adults [38^▪^] reported a substantially lower lifetime prevalence rate [0.5% (Standard Error 0.06)] in females. Possible explanations for this low rate are the use of lay interviewers rather than clinicians and the use of a questionnaire that has not been validated for eating disorder diagnoses. The combination of these factors could lead to underreporting, especially in the case of bulimia nervosa where stigma and shame around bingeing and purging play an important role.

Few recent studies reported on prevalence in males. The results were in the same order as found in the systematic reviews [28,29], with lifetime prevalence rates ranging between 0.1% and 1.2% [11^▪^,38^▪^,^47^^▪▪^]. In males like in females [36^▪▪^,46^▪^], bulimia nervosa does occur in all age groups up to the age of 80, although the prevalence declines after age 30 [45].

The preliminary evidence for an increase in prevalence of bulimia nervosa in population-based studies is noteworthy in the face of decreasing incidence rates in primary and secondary care. One possible explanation is that people who have a lower frequency of binge eating and compensatory behaviour (meeting DSM-5 but not DSM-IV criteria, i.e. once a week) seek help less often than those who have this behaviour more often, and thus are less often included in care-based studies. Future research is needed to clarify this apparent discrepancy.

### Mortality

Mortality could be described as an incidence rate in which the event being measured is death. The crude mortality rate (CMR) is the number of deaths within the study population over a specified period. The standardized mortality ratio (SMR) is the ratio of observed deaths in the study population versus that of expected deaths in the population of origin. For comparison reasons the SMR is preferred, because the CMR is not standardized for age and sex. Mortality is often used as an indicator of the severity of a disorder.

In a previous review in this journal [27], it has been reported that both anorexia nervosa and bulimia nervosa were associated with significantly increased mortality rates. In comparison to age-matched and sex-matched people in the general population, the mortality risk was around two times higher in people followed up after outpatient treatment for anorexia nervosa, or after any treatment for bulimia nervosa. In people followed up after inpatient treatment for anorexia nervosa, the mortality risk was even over five times higher. Since this previous review, a few new studies have been published.

In a landmark meta-analysis of worldwide eating disorder mortality rates by Arcelus *et al.*[50] the CMR of anorexia nervosa patients was 5.1 deaths per 1000 person-years (95% CI 4.0–6.1). The SMR was 5.9 (95% CI 4.2–8.3), i.e. an almost 6 times increased risk. In a recent study, after 5 years follow-up the SMR of anorexia nervosa inpatients with (complications of) severe malnutrition was found to be as high as 15.9 (95% CI 11.6–21.4) [51]. This study population was probably more severely affected than most of the study populations included in the meta-analysis [50]. For bulimia nervosa, Arcelus *et al.* reported a CMR of 1.7 per 1000 person-years (95% CI 1.1–2.4) and a SMR of 1.9 (95% CI 1.4–2.6) [50]. Recently, in a large 12-year follow-up study a higher mortality risk was found in females after inpatient treatment for bulimia nervosa compared to similar-age females hospitalized for pregnancy-related events [adjusted hazard ratio 4.7 (95% CI 2.1–10.8)] [52]. This difference could be even larger when compared to the general population. Iwajomo *et al.* investigated mortality after hospitalization for an eating disorder (anorexia nervosa, bulimia nervosa or eating disorder not otherwise specified) in a Canadian population-based cohort [53^▪^]. Although results were not presented for each eating disorder separately, the total SMR was five times higher compared to the general population [SMR 5.1 (95% CI 4.8–5.3)]. Rates were higher for males [SMR 7.2 (95% CI 6.6–8.0)] compared to females [SMR 4.6 (95% CI 4.3–4.9)]. This is in line with other recent studies that also found higher mortality rates among males: in people treated for bulimia nervosa in secondary mental healthcare services an overall SMR of 2.5 (95% CI 1.5–4.0) was reported, with significantly higher rates among males compared to females [crude hazard ratio 5.4 (95% CI 1.8–16.5)] [54]. In another study on hospitalization for anorexia nervosa, in-hospital mortality in males was more than twice that for females [odds ratio 2.4 (95% CI 1.5–3.8)] [55]. However, in a study directly comparing males and females from the same hospital, from the same treatment period and for the same follow-up period, no significant differences in SMR were found for anorexia nervosa or for bulimia nervosa [56^▪^], which might be due to the relatively small sample size of males. The SMRs for each sex-group [57,58] have already been described in the previous review [27]. While there were no significant differences in mortality rates, males with anorexia nervosa or bulimia nervosa did die sooner in comparison to females with anorexia nervosa or bulimia nervosa [56^▪^].

In summary, recent findings accentuate high mortality rates for anorexia nervosa and bulimia nervosa, with highest rates among those who received inpatient treatment for anorexia nervosa. Although results are still inconclusive, the suggestion that males have a probably higher mortality risk than females underscores the clinical relevance of detecting and treating anorexia nervosa and bulimia nervosa in males.

## CONCLUSION

Anorexia nervosa and bulimia nervosa occur among females and males of all age groups worldwide and are associated with an increased mortality risk. The trend of a decreasing peak age at incidence has implications for future research into risk factors, the development of earlier prevention programs and planning of treatment services. Besides the well-known risk group of young females from Western countries, the occurrence of anorexia nervosa and bulimia nervosa in males, older persons and non-Western countries highlights the need for further research in these groups. Moreover, improved awareness will lead to earlier detection and treatment in these groups that suffer from an extra stigma of a ‘young, Western, female-specific’ psychiatric disorder.

## Acknowledgements

*The authors would like to thank Judith Offringa for her editorial assistance.*

### Financial support and sponsorship

*None.*

### Conflicts of interest

*There are no conflicts of interest.*

## REFERENCES AND RECOMMENDED READING

Papers of particular interest, published within the annual period of review, have been highlighted as:

▪ of special interest
▪▪ of outstanding interest
